# Dietary microalgae *Schizochytrium* spp. and *Nannochloropsis gaditana* modulate antioxidant and immune-related gene expression in ewes’ monocytes and neutrophils

**DOI:** 10.1093/jas/skag165

**Published:** 2026-05-22

**Authors:** Alexandros Mavrommatis, Panagiota Kyriakaki, Rafaela Andreaki, Alexis Skourtis, Eleni Tsiplakou

**Affiliations:** Laboratory of Nutritional Physiology and Feeding, Department of Animal Science, School of Animal Biosciences, Agricultural University of Athens, Athens 11855, Greece; Laboratory of Nutritional Physiology and Feeding, Department of Animal Science, School of Animal Biosciences, Agricultural University of Athens, Athens 11855, Greece; Laboratory of Nutritional Physiology and Feeding, Department of Animal Science, School of Animal Biosciences, Agricultural University of Athens, Athens 11855, Greece; Laboratory of Nutritional Physiology and Feeding, Department of Animal Science, School of Animal Biosciences, Agricultural University of Athens, Athens 11855, Greece; Laboratory of Nutritional Physiology and Feeding, Department of Animal Science, School of Animal Biosciences, Agricultural University of Athens, Athens 11855, Greece

**Keywords:** eicosanoids, redox balance, inflammation, PUFA metabolism, antioxidant enzymes, immune modulation

## Abstract

The inclusion of microalgae in ruminant nutrition offers a well-documented approach to enhance the n-3 polyunsaturated fatty acid (PUFA) profile of animal products while potentially modulating immune and oxidative functions. This study evaluated the long-term effects of dietary supplementation with *Schizochytrium* spp. and *Nannochloropsis gaditana* on the oxidative-inflammatory interplay in dairy ewes. Thirty-two crossbred dairy ewes in early lactation (50 ± 5 d in milk) were divided into four homogeneous groups (*n* = 8 per group) based on milk yield, body weight, and age. The control group (CON) received a concentrate without microalgae supplementation, whereas the treated groups received concentrates supplemented daily with 30 g of *Schizochytrium* spp. (SC30) or with 30 g (MB30) or 40 g (MB40) of a microalgae blend of *Schizochytrium* spp. and *N. gaditana* in a 70:30 ratio. Accordingly, MB30 supplied 21 g of *Schizochytrium* spp. and 9 g of *N. gaditana*, while MB40 supplied 28 g of *Schizochytrium* spp. and 12 g of *N. gaditana*. On the 30th, 60th, and 90th day of the experiment, blood monocytes and neutrophils were isolated to assess the expression of genes involved in antioxidant defense, eicosanoid metabolism, and immune response. Microalgae supplementation did not affect feed intake but altered the dietary fatty acid profile, and increased the intake of long-chain PUFA (DHA, docosahexaenoic acid; n-6 DPA, docosapentaenoic acid; EPA, eicosapentaenoic acid). Moderate supplementation of microalgae blend (MB30) upregulated *GPX1* and *GPX2* expression, while higher inclusion (MB40) enhanced *CAT* transcription but suppressed *SOD2* and *GPX2* in monocytes. Genes associated with eicosanoid synthesis (*PLA2G4A*, *COX*, *LTA4H*, *ALOX5AP*) were significantly downregulated in monocytes, particularly in MB40. Likewise, key immune-related transcripts (*TLR4*, *IFNG*, *IL1B*, *IL2*) were markedly decreased in MB40. Correlation analysis revealed that long-chain PUFA were inversely associated with pro-inflammatory and oxidative genes. Overall, the combined supplementation of *Schizochytrium* spp. and *N. gaditana* modulated redox-sensitive and immune signaling pathways, suggesting a transcriptional profile consistent with reduced expression of pro-inflammatory genes in ewes.

## Teaser

A microalgae diet rich in n-3 fatty acids altered immune cell gene expression in dairy sheep, with changes related to antioxidant and inflammatory pathways, without affecting feed intake.

## Introduction

The immune system functions as a central regulatory network integrating metabolism, oxidative balance, and host defense against both environmental and endogenous stressors (Medzhitov 2008; Radzikowska et al. 2019). In high-producing ruminants bred under intensive farming systems, the intense metabolic demands associated with elevated productivity can trigger chronic low-grade inflammation, which compromises fertility, welfare, and overall performance (McGrath et al. 2018). Both the innate and adaptive immune systems are coordinated through a complex network of cytokines and bioactive lipid mediators, such as eicosanoids. Under standard feeding conditions, eicosanoids are primarily derived from arachidonic acid (ARA) via cyclooxygenase enzymes, generating pro-inflammatory prostaglandins and thromboxanes (Calder 2006). In contrast, long-chain n-3 polyunsaturated fatty acids (PUFAs), including eicosapentaenoic acid (EPA) and docosahexaenoic acid (DHA), and n-6 docosapentaenoic acid (DPA), serve as substrates for the synthesis of specialized pro-resolving mediators, such as resolvins, protectins, and maresins, which exert anti-inflammatory or inflammation-resolving properties (Calder 2006; Nauroth et al. 2010). For example, lipopolysaccharide (LPS) stimulation of Toll-like receptor 4 (TLR4) on innate immune cells activates the NF-κB and mitogen-activated protein kinase (MAPK) cascades, driving transcription of tumor necrosis factor alpha (TNF-α) and interleukin-1β (IL-1β). Notably, n-3 PUFA have been shown to attenuate this signaling cascade and blunt excessive cytokine expression (Calder 2006; Lingappan 2018).

Microalgae have emerged as valuable feed additives in ruminant nutrition, providing a rich source of long-chain PUFAs, antioxidants, and essential micronutrients (Mavrommatis et al. 2023; El-Sayed et al. 2024). Among them, *Schizochytrium* spp. are rich in DHA and DPA, whereas *Nannochloropsis* spp. are prominent sources of EPA (El-Sayed et al. 2024). Beyond their bioactive lipid fraction, *Nannochloropsis* are rich in a variety of other bioactive compounds, such as flavonoids, β-carotene, phenolic acids, phycocyanins, steroids, saponins, chlorophyll, and triterpenoids, that also exert antioxidant, anti-inflammatory, and immunostimulatory activities (Emam et al. 2024).

DHA-rich lipids such as those derived from microalgae *Schizochytrium* have been demonstrated to mitigate excessive inflammatory responses, largely through the inhibition of TLR4/NF-κB signaling in macrophages (Oh et al. 2010) and monocytes in goats (Mavrommatis et al. 2021a). It is worth noting that different immune cell types display variable transcriptional responses to n-3 PUFA due to differences in membrane composition and receptor signaling (Radzikowska et al. 2019). Another critical aspect of PUFA-rich microalgae supplementation in ruminant diets lies in the interplay between pro-oxidant production and antioxidant defense mechanisms. Algal long-chain PUFA, due to their high degree of unsaturation, are particularly prone to lipid peroxidation, which may increase reactive oxygen species (ROS) formation (Di Nunzio et al. 2016). In turn, excessive ROS production can damage cellular components and amplify inflammation via oxidative activation of NF-κB and related transcription factors (Andrés et al. 2023). Therefore, the antioxidant-prooxidant interplay plays a pivotal role in determining whether dietary PUFA-rich microalgae exerts a protective or adverse immuno-oxidative effect. Previous evidence in dairy goats shows that high dietary inclusion of microalgae *Schizochytrium* spp. elevated blood NADPH oxidase activity and oxidative biomarkers, indicating increased oxidative burst (Mavrommatis et al. 2018). Controlled modulation of oxidative pathways is thus essential to achieve immunological benefits without inducing oxidative damage.

Although the immunomodulatory properties of microalgal PUFA are well documented, scarce evidence exists in livestock, apart from our previous experiments focused specifically on *Schizochytrium* spp. (Mavrommatis et al. 2021a; Kyriakaki et al. 2023) and other studies conducted in marine organisms (Habte-Tsion et al. 2020; El-Hawy et al. 2022a, 2022b; Zahran et al. 2023; Ardó et al. 2025). Considering these aspects, combining DHA/DPA-rich *Schizochytrium* spp. with EPA-rich *Nannochloropsis gaditana* represents a promising nutritional strategy to enhance immune-oxidative resilience in dairy sheep. Their complementary fatty acid profiles and other bioactive compounds could synergistically expand the spectrum of pro-resolving lipid mediators while simultaneously sustaining or even improving antioxidant protection. Therefore, the objective of the present study is to evaluate the effects of dietary supplementation with *Schizochytrium* spp. and their combination with *Nannochloropsis gaditana* on the expression of genes involved in the immune-oxidative interplay in blood monocytes and neutrophils of dairy sheep.

## Material and methods

Animal handling procedures were performed following protocols approved by the Agricultural University of Athens Ethical Committee (Number approval; 31/27-05-2021). The study was conducted with respect to the guidelines of the European Union Directive on the defense of animals used for scientific purposes following directive EU 63/2010 and Council of the European Union 2010.

### Animals and diets

Thirty-two crossbred dairy ewes [Lacaune × Local (Greek) breed] of comparable age (3–4 yr old) and body weight (74 ± 4 kg), at early lactation (50 ± 5 d in milk) were used. The ewes were separated into four homogenous groups (*n* = 8 per group) according to their milk yield (Fat Corrected Milk, FCM 6%; 2.73 ± 0.433 kg), body weight, and age.

Each ewe was offered 2 kg of alfalfa hay and 2 kg of concentrate daily (F:C = 50:50), divided into two equal meals (08:00 and 20:00 h), and was fed individually throughout the experimental period. NRC (2007) was used to predict the nutrient requirements of dairy ewes. The concentrates supplied to the control (CON) group had no microalgae while those of the treated groups were supplemented daily with 30 g microalgae *Schizochytrium* spp. (SC30) and 30 (MB30) and 40 (MB40) g with microalgae blend of *Schizochytrium* spp./*N. gaditana* into a portion 70/30 (MB30: 21 g *Schizochytrium* spp. and 9 g *N. gaditana*; MB40: 28 g *Schizochytrium* spp. and 12 g *N. gaditana*). Microalgae supplementation levels were selected based on previous dose-response studies in sheep (Zisis et al. 2022) and goats (Mavrommatis et al. 2018). Additionally, the proportion of the two microalgae was selected based on preliminary unpublished data investigating the minimum EPA inclusion rate from *N. gaditana* required to exert a substantial effect on milk fatty acid profile. Microalgae were incorporated into the concentrate by partially replacing soybean oil and palm oil in the control (CON) formulation, in order to achieve isoenergetic diets with comparable ether extract levels, without markedly altering the overall ingredient composition (Supplementary Table S1). Refusals were recorded daily and pooled weekly for chemical analysis to determine the actual nutrient intake after forage sorting. The *Schizochytrium* spp. used was a commercial product (DHA GOLD S17-B, DSM Nutritional Products, Marousi, Greece) while *N. gaditana* was cultivated by Mikrophykos, Athens, Greece. The concentrate diets were prepared every 20 d and kept under room temperature conditions avoiding sunlight in order to prevent any oxidation of the microalgae. The experimental period lasted 100 d, with the first 10 d considered an adaptation period for the animals in the pens and feeders. Thus, the main experimental period was 90 d.

Samples of microalgae and alfalfa hay were collected prior to the experimental period for nutritional assessment, while concentrate samples were collected every 20 d, coinciding with the preparation of new batches. Refusals were collected as described above and analyzed at the end of the trial. Samples were analyzed for crude protein (CP; Official Method 7.016) according to the Association of Official Analytical Chemists (AOAC 1984) using a Kjeldahl Distillation System (FOSS Kjeltec 8400, Hillerød, Demark) while ether extract (EE) was determined according to AOAC Official Method 920.39. The dry matter (DM) was measured by drying the sample at 103°C overnight (7.007) according to AOAC (1984). The neutral detergent fiber (aNDF) in alfalfa hay and concentrates were analyzed according to AOAC Official Method 2002.04 described by Mertens (2002), while acid detergent fiber (ADF) according to AOAC Official Method 973.18 (AOAC 2005). The aNDF was determined using heat-stable amylase but no sodium sulfite and expressed inclusive of the residual ash. Feeds were also analyzed for fatty acids composition according to the method of O’Fallon et al. (2007) using an Agilent 6890 N gas chromatograph equipped with an HP-88 capillary column (60 m × 0.25 mm i.d. with 0.20 µm film thickness, Agilent Technologies, Inc., Santa Clara, CA, USA) and a flame ionization detector (FID) as described by Mavrommatis et al. (2021b). The FID temperature was set at 260°C, and the chromatographic analysis involved a temperature-programmed run starting at 120°C and held for 1 min. Then, the ramp was followed by two steps: one step of 1.25°C/min to 230°C and another step of 10°C/min to 240°C and held for 3 min. Hydrogen was used as the carrier gas with a linear velocity set at 30 cm/s, and helium was the make-up gas. Each peak was identified and quantified using a 37 component FAME mix standard (Supelco, Sigma-Aldrich Co., St. Louis, MO, USA). Additionally, PUFA No.1 Marine source, analytical standard (47033; Supelco, Sigma-Aldrich Co., St. Louis, MO, USA) was used for identify microalgae long chain PUFA. Tridecanoic acid (C13:0) was used as an internal standard for chromatographic analysis (Fluka, Sigma-Aldrich Co.).

### Cell isolation, RNA extraction, and cDNA synthesis

Twenty ml of blood samples were collected from the jugular vein into 17 units/ml heparin-containing tubes, on the 30th, 60th, and 90th day from the beginning of the experiment. For monocytes and neutrophils isolation, Histopaque density gradient (Sigma-Aldrich Co., USA) was used, as described by Tsiplakou et al. (2018). Total RNA was extracted from approximately 5 × 10^6^ cells using peqGOLD TriFast (VWR, International) according to the manufacturer’s instructions. Five μg of each extracted RNA were treated with Turbo DNAse using a commercial kit (Invitrogen, California, USA). After DNase treatment, RNA was correlated with a positive control (ewe’s genomic DNA) as a template, using glyceraldehyde 3-phosphate dehydrogenase (*GAPDH*) primers and a qualitative Taq polymerase PCR protocol to investigate the absence of DNA contamination in agarose gel. Agarose gel (3%) was used for the evaluation of RNA integrity. Discrete bands were monitored in all samples representing 28s and 18s ribosomal RNAs respectively, showing little or no RNA hydrolysis. The quantity of RNA was assessed spectrophotometrically using Nanodrop. Total RNA was reversely transcribed with the PrimeScript First Strand cDNA Synthesis Kit (Takara Japan), according to the manufacturer’s instructions using a mix of random hexamers and oligodT primers.

### Primer design and real-time quantitative PCR

The design of primer pairs was specific for *Ovis aries* species, according to their coding sequence (CDS by GenBank) using PerlPrimer software (Table 1). Relative expression levels of mRNA for target genes were quantified with a Tianlong Gentier96 real-time PCR. PCR cycling started at 95°C for 15 min, followed by 40 cycles of 95°C for 15 s and 60°C to 62°C for 1 min (based on primer pair). Primers’ specificity and formation of primer dimers were also examined by melt curve analysis. Based on Vorachek et al. (2013), the expression levels of *GAPDH* and tyrosine 3-monooxygenase/tryptophan 5-monooxygenase activation protein zeta (*YWHAZ*) were used as housekeeping genes to normalize cDNA templates. The DDCt method was used for the calculation of relative expression levels of target genes, according to Hellemans et al. (2007), while primer efficiency was calculated according to target genes standard curves. Relative gene transcript levels were normalized to the CON group, whose mean value was set to 1.

**Table 1:** Sequences, amplicon size, annealing temperature, and RefSeq number of primers used in real-time qPCR.

Gene	Sequence	Amplicon size	Tm ^o^C	GenBank RefSeq
***Glyceraldehyde-3-phosphate dehydrogenase (GAPDH)***	F: 5′-AAAGGCCATCACCATCTTCCA-3′	75	62	XM_005680968.3
	R: 5′-ACCACGTACTCAGCACCAGCAT-3′			
***Tyrosine 3-monooxygenase/tryptophan 5-monooxygenase activation protein zeta (YWHAZ)***	F: 5′-TGTTCTATTGTGCCTAGTACACTGT-3′	70	62	XM_018058314.1
	R: 5′-CATCAAGACTCACTGCCTCCC-3′			
***Catalase (CAT)***	F: 5′-GAGGAAACGCCTGTGTGAGA-3′	116	60	XM_005690077.3
	R: 5′-GGATGCGGGAGCCATATTCA-3′			
***Superoxide dismutase 1 (SOD1)***	F: 5′-ATCCACTTCGAGGCAAAGGG-3′	124	60	NM_001285550.1
	R: 5′-CTGCACTGGTACAGCCTTGT-3′			
***Superoxide dismutase 2 (SOD2)***	F: 5′-GCCCGATTATCTGAAGGCCA-3′	99	60	XM_018053428.1
	R: 5′-CTCAGTGTAAGGCTGACGGT-3′			
***Glutathione peroxidase 1 (GPX1)***	F: 5′-CATCGACATCGAGCCTGACA-3′	109	60	XM_005695962.3
	R: 5′-AAAATCCCCGGAGAGCAGTG-3′			
***Glutathione peroxidase 2 (GPX2)***	F: 5′-CCTCCCCACCCCTTTAATCG-3′	115	62	XM_005685982.3
	R: 5′-GGCTGATAGCACTGAGGTCG-3′			
***Glutathione peroxidase 3 (GPX3)***	F: 5′- GGAGGCCAAGGGGAAGTAAC-3′	114	60	XM_005683183.3
	R: 5′-GCATGGGAGTGTGGCATAGT-3′			
***Glutathione transferase 2 (GMST2)***	F: 5′-AAAGTTATGCCCCCATCCGT-3′	85	60	XM_013970519.2
	R : 5′-CACCAGACCCAGACAAGTAGC-3′			
***Glutathione transferase 3 (GMST3)***	F: 5′-CCCCACTCTGATAGAGGCCA-3′	121	60	XM_013975063.2
	R : 5′-GTAGTCGTCCAGCCTCGTTT-3′			
***Nicotinamide adenine dinucleotide phosphate oxidase 1; (NOX1)***	F: 5′-TCTTTCAAGCCTCGAGTCCC-3′	74	60	XM_018044365.1
	R: 5′-AGGTCCATGAAGCTCAGTGATG-3′			
***Nicotinamide adenine dinucleotide phosphate oxidase 2 (NOX2)***	F: 5′-ACGACCCAACTGGGATAACG-3′	127	60	XM_005700924.3
	R: 5′-GGAGTTGGAGATGCACTGCT-3′			
***Cyclooxygenase-2 (COX2)***	F: 5′-TCCCATCCATGCCAGAATCG-3′	77	60	XM_018060731.1
	R: 5′-CCTGTTCGGGTACAGTCACA-3′			
***Prostaglandin E receptor 2 (PTGER2)***	F: 5′-GGACACAAGCAGACCACGTA-3′	108	60	NM_001314255.1
	R: 5′-CATGCGGATGAGGTTGACGA-3′			
***Arachidonate 12-Lipoxygenase (ALOX12)***	F: 5′-AGGACTGCGCTCAAATCAGG-3′	83	60	XM_018064507.1
	R: 5′-TCCTGGAGAGTGGGCTTCTC-3′			
***Arachidonate 5-Lipoxygenase Activating Protein (ALOX5AP)***	F: 5′-ACTTTGTTGGCTACCTGGGG-3′	107	60	XM_005687536.3
	R: 5′-GTTGAGTATCCCAGCGAGGG-3′			
***Leukotriene A4 Hydrolase (LTA4H)***	F: 5′-TCCCTTTCTCTCGCGCTCAG-3′	78	61	XM_005680471.3
	R : 5′-GTGAGGAGTCCCGATGCAC-3′			
***Leukotriene C4 Synthase (LTC4S)***	F: 5′-TGTCTAGGGCTGGAGGAAAG-3′	102	60	XM_018051605.1
	R: 5′-CAGAAGTACCAGGGAGCAGATG-3′			
***Cytosolic phospholipase A2 (PLA2G4A)***	F: 5′-TTGTGCTACAGAGAGGAGAGGA-3′	119	61	XM_018060732.1
	R: 5′-GTGCCACGTAGCACCACTAC-3′			
***Toll like reseptors 4 (TLR4)***	F: 5′-ATGAACCACTCCACTCGCTC-3′	70	62	NM_001135930.1
	R: 5′-TCTTGCTCCTTAGAGGCCGT-3′			
***Mitogen-activated protein kinase 1 (MAPK)***	F: 5′-GCAACGACCACATCTGCTAC-3′	100	62	XM_027956867.2
	R: 5′-AGGTTGGAAGGCTTGAGGTC-3′			
***Interferon gamma (IFNG)***	F: 5′-AAATTCCGGTGGATGATCTG-3′	146	60	NM_001009803.1
	R: 5′-ACCATTACATTGATGCTCTCC-3′			
***Interleukin 1 beta (IL1B)***	F: 5′-TGGATAGCCCATGTGTGCTG-3′	70	62	NM_001009465.2
	R: 5′-CAGAACACCACTTCTCGGCT-3′			
***Interleukin 2 (IL2)***	F: 5′-GGAAGTGCTAGATTTAGCTCCA-3′	105	60	NM_001009806.1
	R: 5′-GTTTCAGATCCCTGTAGTTCCA-3′			
***Interleukin 10 (IL10)***	F: 5′-CTGGGGGAGAAGCTGAAGAC-3′	100	62	NM_001009327.1
	R: 5′-CTCTCTTCACCTGCTCCACC-3′			
***Tumor necrosis factor alpha (TNFA)***	F: 5′-CGTTGTAGCCAACATCAGC-3′	143	60	NM_001024860.1
	R: 5′-GGACCTGCGAGTAGATGAG-3′			
***Nuclear factor kappa B (NFkB)***	F: 5′-ACAAATAGACGAGCTCCAGG-3′	123	60	XM_060416845.1
	R: 5′-GGCACTTTGTTAAGAGTTAGCA-3′			
***Chemokine (C-C motif) ligand 5 (CCL5)***	F: 5′-CAAGTGCTCCATGGCAGCAG-3′	61	62	XM_027975305.3
	R: 5′-GTTGGCGCACACCTGACG-3′			
***C-X-C motif chemokine ligand 16 (CXCL16)***	F: 5′-GTGCCTGTGTTGTCCCTCTT-3′	70	62	XM_015098600.3
	R: 5′-GCTTGCACACCACGTAGAGT-3′			

### Statistical analysis

Data were analyzed using IBM SPSS Statistics (version 26.0) and are presented as least squares means (LS-means) with their standard errors (SE) in bar graphs. Dietary group effects were tested using a general linear model for repeated measures analysis of variance with dietary groups (D = CON, SC30, MB30, MB40) as a fixed factor and the sampling times (S = 30th, 60th, and 90th experimental day) as the repeated measure and their interactions (D × S). Post hoc analysis was executed using the Tukey test while the significance threshold was set at 5%, whilst results with 0.05 ≤ *P *< 0.10 were considered to indicate a statistical tendency. The effects of dietary group, sampling time, and their interaction are provided in Supplementary Tables S3 and S4. Discriminant analyses were also applied to pooled gene expression data to identify the variables capable of discriminating and classifying samples among the four dietary groups, using Wilks’ lambda (Λ) criterion. Separate models were developed for monocytes, neutrophils, and the combined dataset (monocytes + neutrophils). In addition, principal component analysis (PCA) was conducted to visualize the dimensional distribution of monocyte and neutrophil responses based on the examined variables.

## Results

### Feed intake

Chemical characterization demonstrated clear differences in the composition of the two microalgal sources (Supplementary Table S2). *Schizochytrium* spp. was distinguished by a greater ether extract content and a notably high proportion of docosahexaenoic acid (DHA; C22:6 n-3). In contrast, *N. gaditana* showed higher crude protein levels and was primarily enriched in eicosapentaenoic acid (EPA; C20:5 n-3). The daily nutrient intake of sheep receiving the experimental diets is summarized in Table 2. No significant dietary effects were observed on the intake of dry matter, ash, crude protein, acid detergent fiber, or neutral detergent fiber (*P *> 0.05). However, ether extract intake varied significantly among treatments (*P *< 0.001). With respect to fatty acid intake, dietary supplementation with microalgae significantly altered the consumption of most individual fatty acids (Table 2). Significant differences among treatments were detected for myristic (C14:0), palmitic (C16:0), oleic (cis-9 C18:1), linoleic (C18:2 n-6), eicosapentaenoic (C20:5 n-3), docosapentaenoic (C22:5 n-6), and docosahexaenoic (C22:6 n-3) acids. Animals fed the control diet consumed higher amounts of the predominant saturated, monounsaturated, and polyunsaturated fatty acids (C16:0, cis-9 C18:1, and C18:2 n-6) relative to those receiving microalgae-based diets (*P *< 0.001). Conversely, supplementation with *Schizochytrium* spp. or *N. gaditana* substantially enhanced the intake of long-chain polyunsaturated fatty acids. Specifically, intakes of C20:5 n-3, C22:5 n-6, and C22:6 n-3 increased progressively with higher inclusion rates of microalgae (*P *< 0.001), reaching maximum levels in the MB40 treatment.

**Table 2:** Nutrient daily intake of the experimental diet throughout the experimental period.

	Dietary treatment^1^					
Item (in g·d^−1^ except as noted)	CON	SC30	MB30	MB40	SEM^2^	*P*-value
** DM**	3404.2	3458.6	3432.1	3473.0	10.478	0.102
** Ash**	248.9	250.5	250.7	253.4	0.689	0.142
** CP**	599.1	603.5	600.4	607.0	1.246	0.101
** Ether extract**	137.6^a^	135.2^b^	132.5^c^	135.4^b^	0.349	<0.001
** aNDF**	1142.0	1126.0	1134.0	1138.0	6.003	0.191
** ADF**	693.0	705.4	702.5	718.6	4.187	0.192
Fatty acids intake in g·d^−1^						
** C14:0**	2.67^a^	3.55^b^	3.29^c^	3.54^b^	0.156	0.001
** C16:0**	49.29^a^	46.82^b^	46.33^c^	46.89^b^	2.394	<0.001
** C18:0**	4.80	4.30	4.28	4.29	0.220	0.108
** cis-9 C18:1**	18.40^a^	13.90^b^	13.94^b^	13.95^b^	0.777	<0.001
** C18:2 n-6**	46.24^a^	43.89^b^	43.89^b^	43.89^b^	2.224	<0.001
** C18:3 n-3**	13.37	13.54	13.46	13.58	0.669	0.101
** C20:5 n-3**	0.03^c^	0.03^c^	0.34^b^	0.44^a^	0.011	<0.001
** C22:5 n-6**	0.02^a^	1.15^a^	0.81^c^	1.08^b^	0.038	<0.001
** C22:6 n-3**	0.00^d^	3.64^a^	2.55^c^	3.39^b^	0.120	<0.001

1 CON: concentrate mix without microalgae; SC30: 30 g microalgae *Schizochytrium* spp.·animal^−1^·d^−1^; MB30: 30 microalgae blend g·animal^−1^·d^−1^ (21 g *Schizochytrium* spp. and 9 g *Nannochloropsis gaditana*); MB40: 40 microalgae blend g·animal^−1^·d^−1^ (28 g *Schizochytrium* spp. and 12 g *Nannochloropsis gaditana*).

2 SEM = Standard error of the mean.

Different lowercase superscript letters (a, b, c, d) indicate significant differences (*P *< 0.05) among dietary treatments.

### Antioxidant/pro-oxidant-related gene expression

**Figure 1:**
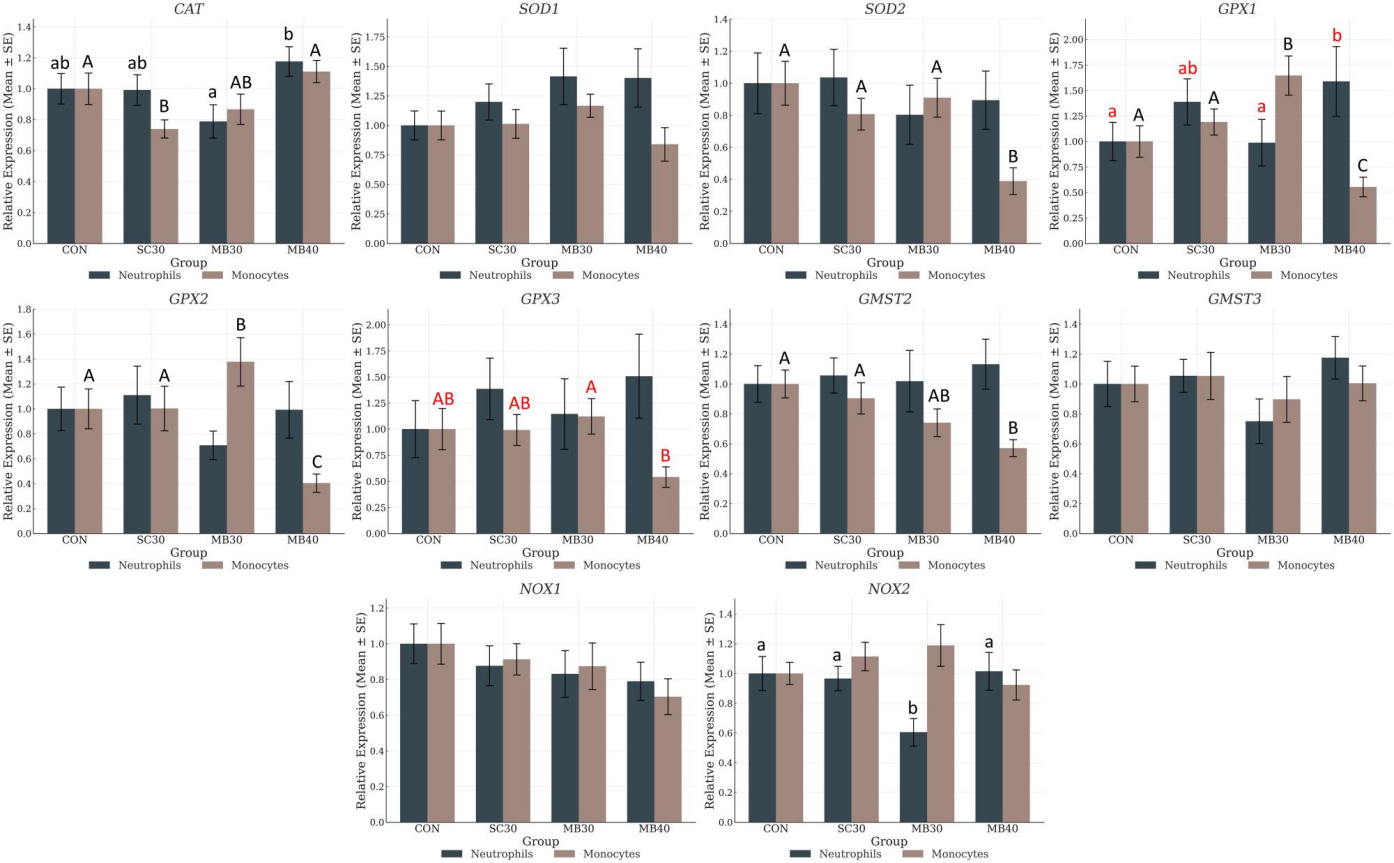
Relative transcript levels of antioxidants/prooxidants-related genes in neutrophils (blue bars) and monocytes (brown bars) of ewes fed CON, SC30, MB30, and MB40 diets for 90 d. Different lowercase superscript letters (a, b) indicate significant differences (*P* < 0.05) among treatments in neutrophils, while different uppercase superscript letters (A, B, C) indicate significant differences (*P *< 0.05) among treatments in monocytes. Black superscript letters indicate statistical significance (*P *< 0.05), whereas red superscript letters denote a statistical trend (0.05 ≤ *P *< 0.10). CON: concentrate mix without microalgae; SC30: 30 g microalgae *Schizochytrium* spp.·animal^−1^·d^−1^; MB30: 30 microalgae blend g·animal^−1^·d^−1^ (21 g *Schizochytrium* spp. and 9 g *Nannochloropsis gaditana*); MB40: 40 microalgae blend g·animal^−1^·d^−1^ (28 g *Schizochytrium* spp. and 12 g *Nannochloropsis gaditana*).

Dietary supplementation with microalgae significantly affected several genes associated with the antioxidant defense system and pro-oxidant cascade in both neutrophils and monocytes (Figure 1). In neutrophils, the expression of *CAT* (*P *= 0.028) was increased in ewes receiving the MB40 diet compared with MB30, whereas the SC30 and CON remained unaffected. A similar pattern was evident in monocytes (*P *= 0.028), where MB40-fed ewes displayed greater *CAT* transcription than SC30. The mRNA abundance of *GPX1* in neutrophils tended to be affected by treatment (*P *= 0.066), being elevated in MB40 compared with CON and MB30. In monocytes, both *GPX1* and *GPX2* were upregulated (*P *< 0.001), with the highest expression observed in MB30 and the lowest in MB40. *GPX3* expression in monocytes showed a tendency for reduction in MB40 relative to MB30 (*P *= 0.094). In monocytes, *GMST2* expression was decreased with increasing microalgae supplementation (*P *= 0.049), with MB40 showing lower transcription compared with CON and SC30. The transcription of *SOD2* in monocytes was significantly downregulated (*P *= 0.001) in MB40 compared with all other groups. Among the pro-oxidant genes, neutrophil *NOX2* expression was downregulated (*P *= 0.020) in MB30 compared with CON, SC30, and MB40. NOX1 was not affected (*P *> 0.10) in either cell type.

### Eicosanoid metabolism-related genes

**Figure 2:**
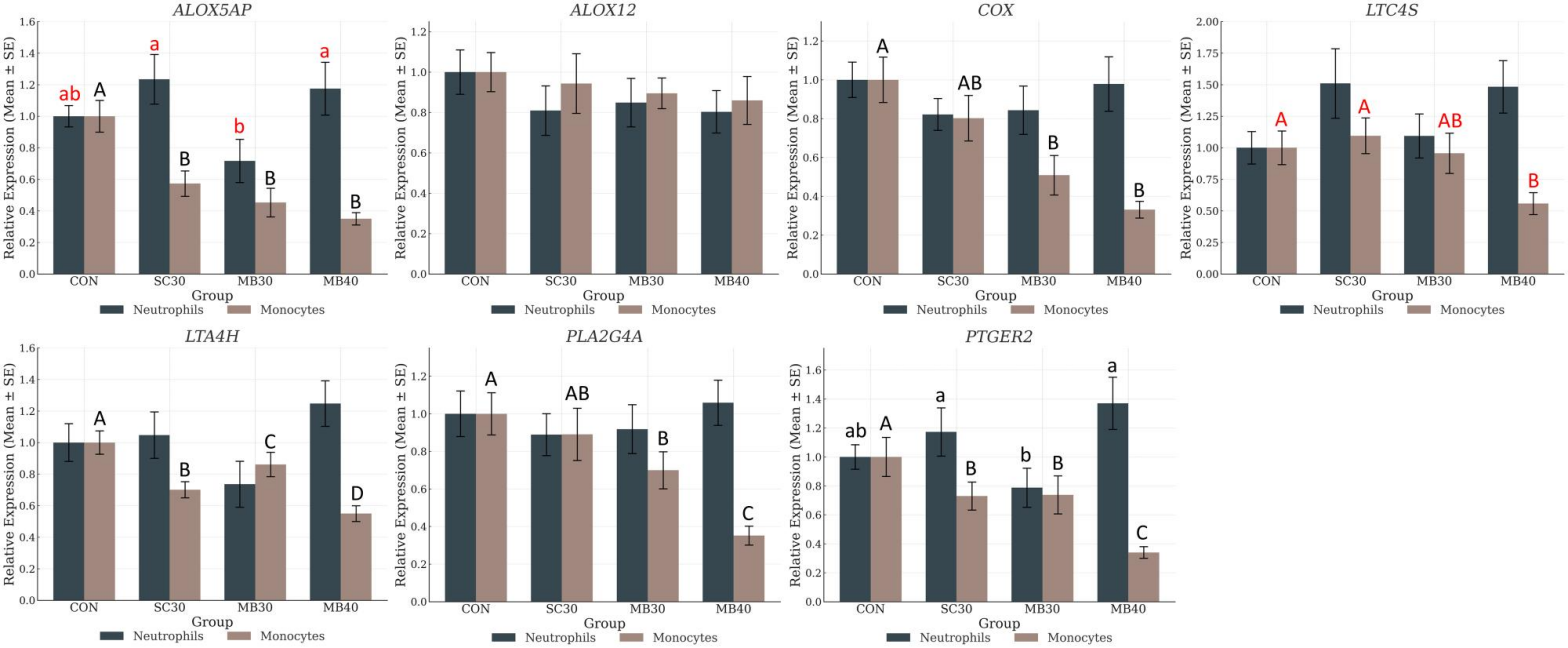
Relative transcript levels of eicosanoids production-related genes in neutrophils (blue bars) and monocytes (brown bars) of ewes fed CON, SC30, MB30, and MB40 diets for 90 d. Different lowercase superscript letters (a, b) indicate significant differences (*P* < 0.05) among treatments in neutrophils, while different uppercase superscript letters (A, B, C) indicate significant differences (*P *< 0.05) among treatments in monocytes. Black superscript letters indicate statistical significance (*P *< 0.05), whereas red superscript letters denote a statistical trend (0.05 ≤ *P *< 0.10). CON: concentrate mix without microalgae; SC30: 30 g microalgae *Schizochytrium* spp.·animal^−1^·d^−1^; MB30: 30 microalgae blend g·animal^−1^·d^−1^ (21 g *Schizochytrium* spp. and 9 g *Nannochloropsis gaditana*); MB40: 40 microalgae blend g·animal^−1^·d^−1^ (28 g *Schizochytrium* spp. and 12 g *Nannochloropsis gaditana*).

Microalgae inclusion modified the expression of genes involved in eicosanoid metabolism and signaling pathways (Figure 2). In neutrophils, *PTGER2* expression was significantly affected (*P *= 0.042), being greater in MB40 compared with MB30, while SC30 and CON remained unaffected. The mRNA abundance of *ALOX5AP* in neutrophils was tended to be downregulated (*P *= 0.098) in MB30 compared to SC30 and MB40. The expression of *ALOX12*, *COX*, *LTC4S*, *LTA4H*, and *PLA2G4A* in neutrophils was not influenced by diet (*P *> 0.10). In monocytes, eicosanoid-related genes displayed consistent downregulation in response to microalgae supplementation. *LTA4H* was markedly reduced (*P *< 0.001) in all supplemented groups, with the lowest expression in MB40. Similarly, *PLA2G4A* (*P *< 0.001), *PTGER2* (*P *= 0.003), *COX* (*P *= 0.002), and *ALOX5AP* (*P *< 0.001) were progressively downregulated with increasing supplementation level and blend combination.

### Immune and inflammatory response gene expression

**Figure 3:**
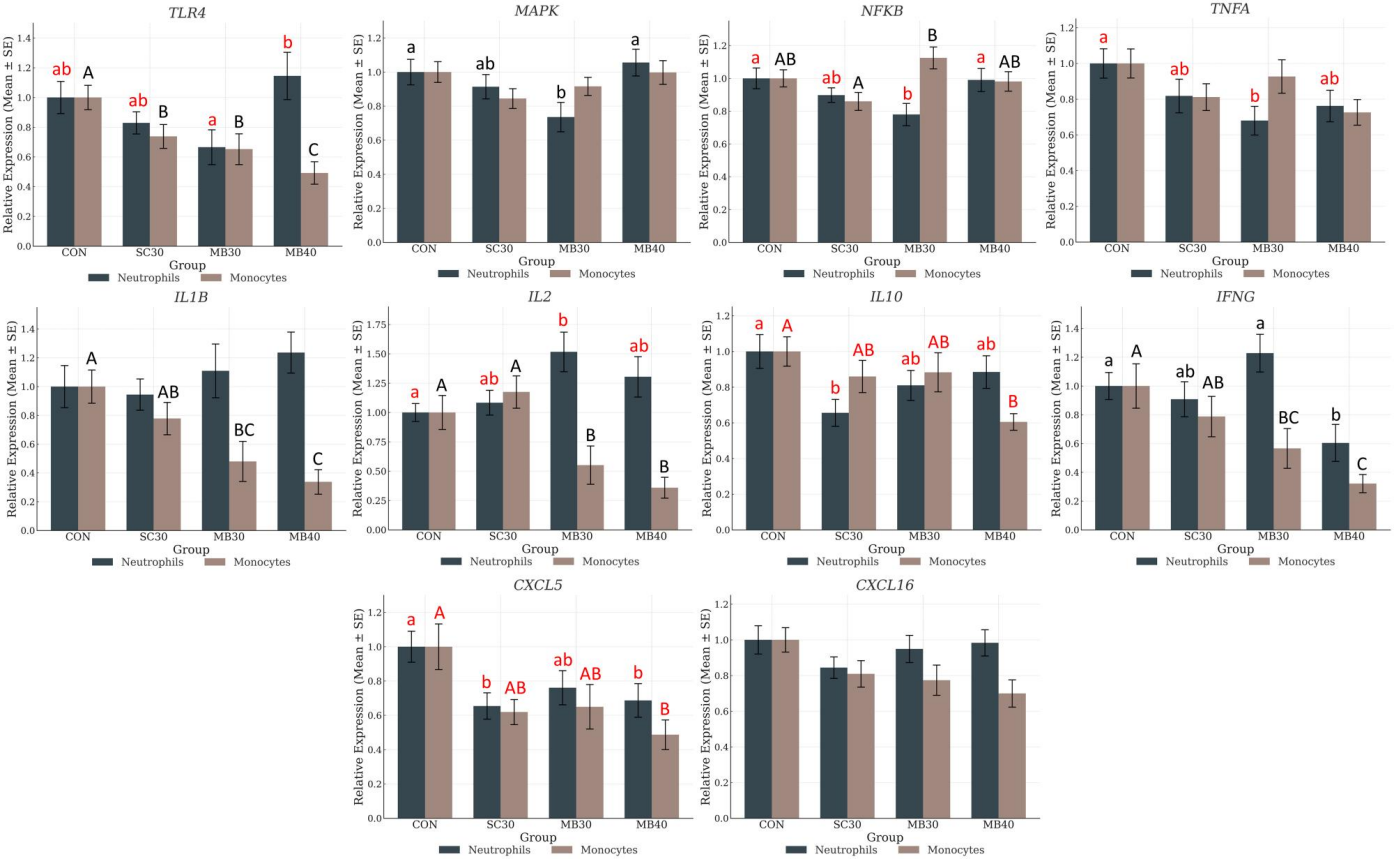
Relative transcript levels of immune-related genes in neutrophils (blue bars) and monocytes (brown bars) of ewes fed CON, SC30, MB30, and MB40 diets for 90 d. Different lowercase superscript letters (a, b) indicate significant differences (*P* < 0.05) among treatments in neutrophils, while different uppercase superscript letters (A, B, C) indicate significant differences (*P *< 0.05) among treatments in monocytes. Black superscript letters indicate statistical significance (*P *< 0.05), whereas red superscript letters denote a statistical trend (0.05 ≤ *P *< 0.10). CON: concentrate mix without microalgae; SC30: 30 g microalgae *Schizochytrium* spp.·animal^−1^·d^−1^; MB30: 30 microalgae blend g·animal^−1^·d^−1^ (21 g *Schizochytrium* spp. and 9 g *Nannochloropsis gaditana*); MB40: 40 microalgae blend g·animal^−1^·d^−1^ (28 g *Schizochytrium* spp. and 12 g *Nannochloropsis gaditana*).

Figure 3 presents changes of immune related gene expression due to dietary treatment in both neutrophils and monocytes. In neutrophils, *MAPK* expression was affected by diet (*P *= 0.046), with MB30 showing lower transcript abundance compared with MB40 and CON while SC30 remained unaffected. *TLR4* tended to vary among groups (*P *= 0.089), being numerically higher in MB40 than MB30. Expression of *IFNG* was downregulated (*P *= 0.024) in MB40 compared with CON and MB30, whereas *NFKB* tended to be lower in MB30 relative to CON and MB40 (*P *= 0.080). *TNFA* tended to be downregulated in MB30 compared to the CON as well (*P *= 0.072). *IL10* tended to be reduced in SC30 compared with CON (*P *= 0.099), and *CXCL5* expression was lower in SC30 and MB40 than in CON (*P *= 0.097). In monocytes, *TLR4* was significantly downregulated by microalgae supplementation (*P *= 0.003), showing the lowest values in MB40. *IFNG* expression followed a similar pattern (*P *= 0.010), being statistically decreased in MB30 and MB40. *NFKB* was affected (*P *= 0.022), with higher transcription in MB30 compared with SC30. *IL1B* was markedly downregulated (*P *= 0.001) in MB30 and MB40 relative to CON, whereas *IL2* downregulated (*P *= 0.001) in MB30 and MB40 compared to both CON and SC30. *IL10* tended to be decreased with increasing supplementation (*P *= 0.098), and *CXCL5* exhibited a similar tendency (*P *= 0.100), both being only numerically lower in MB40 compared with CON.

### Gene expression patterns between monocytes and neutrophils

**Figure 4:**
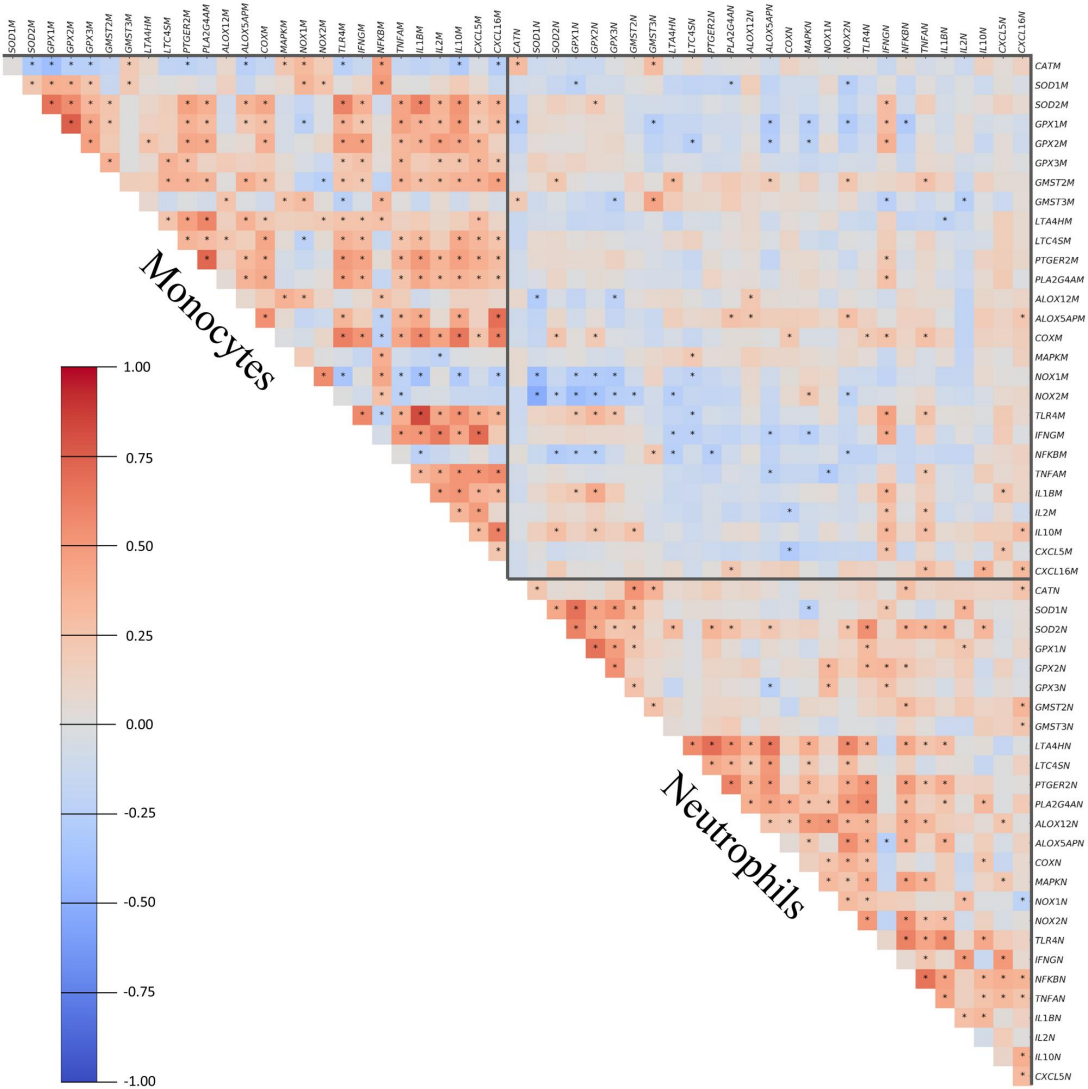
Pearson correlation between monocyte (M) and neutrophil (N) gene expression profiles. Asterisks indicate levels of statistical significance (* *P* < 0.05, ** *P* < 0.01, *** *P* < 0.001).

The correlation heatmap (Figure 4) illustrates the pairwise Pearson correlation coefficients among antioxidant, eicosanoid, and immune‐related genes within and between monocytes and neutrophils. Distinct clustering patterns were evident between the two cell types. Within monocytes, a strong and significant positive correlation network (*P *< 0.05; *R *> 0.70) was observed among genes encoding antioxidant enzymes, including *SOD1*, SO*D2*, *GPX1*, *GPX2*, and *GPX3*, as well as *GSTM2*, and *GSTM3*. These antioxidant markers (*SOD2*, *GPX1*, *GPX2*, *GPX3*, *GSTM2*, and *GSTM3*) were also positively associated with inflammatory and regulatory genes *IL10*, *TLR4*, *IFNG*, *IL2*, *CCL5*, *CXCL16*, and *IL1B* (*P *< 0.05; *R* ≈ 0.45–0.68). At the same time, *NOX1* and *NOX2* showed significant positive correlation with the two principal antioxidant regulators *CAT* and *SOD1* pointing out the response of endogenous antioxidant mechanisms towards pro-oxidant manifestation. Eicosanoid-related genes (*PLA2G4A*, *COX*, *ALOX5AP*, *LTA4H*, *PTGER2*) correlated positively with *TLR4*, *IFNG*, *IL1B*, *IL2*, and chemokines (*R *> 0.60, *P *< 0.05).

In neutrophils, correlations were generally weaker and more functionally compartmentalized compared to monocytes. Moderate-to-strong positive relationships (*R *= 0.50–0.75, *P *< 0.05) were observed within the antioxidant gene set (*CAT*, *SOD1*, *SOD2*, *GPX1–3*, *GSTM2*, *GSTM3*). *NFKB* showed positive correlation with *MAPK* and *TNFA* (*R *= 0.48, *P *< 0.05). In contrast, eicosanoid- and cytokine-related genes presented fewer significant interconnections, indicating fewer significant inter-gene correlations in neutrophils compared with monocytes.

When homologous genes between monocytes and neutrophils were compared, most showed low-to-moderate correlations (*R *< 0.40, *P *> 0.05), indicating largely cell-specific regulatory control. However, modest but significant positive correlations were detected for *TNFA* in neutrophils and *TNFA*, *IL2*, and *IL10* in monocytes, suggesting partial synchronization of inflammatory signaling. Interestingly, *NOX1* and *NOX2* expression in monocytes were strongly and negatively correlated with most antioxidant genes in neutrophils, indicating an inverse association between monocyte pro-oxidant genes and neutrophil antioxidant transcripts.

### Gene expression correlation with fatty acids intake

**Figure 5:**
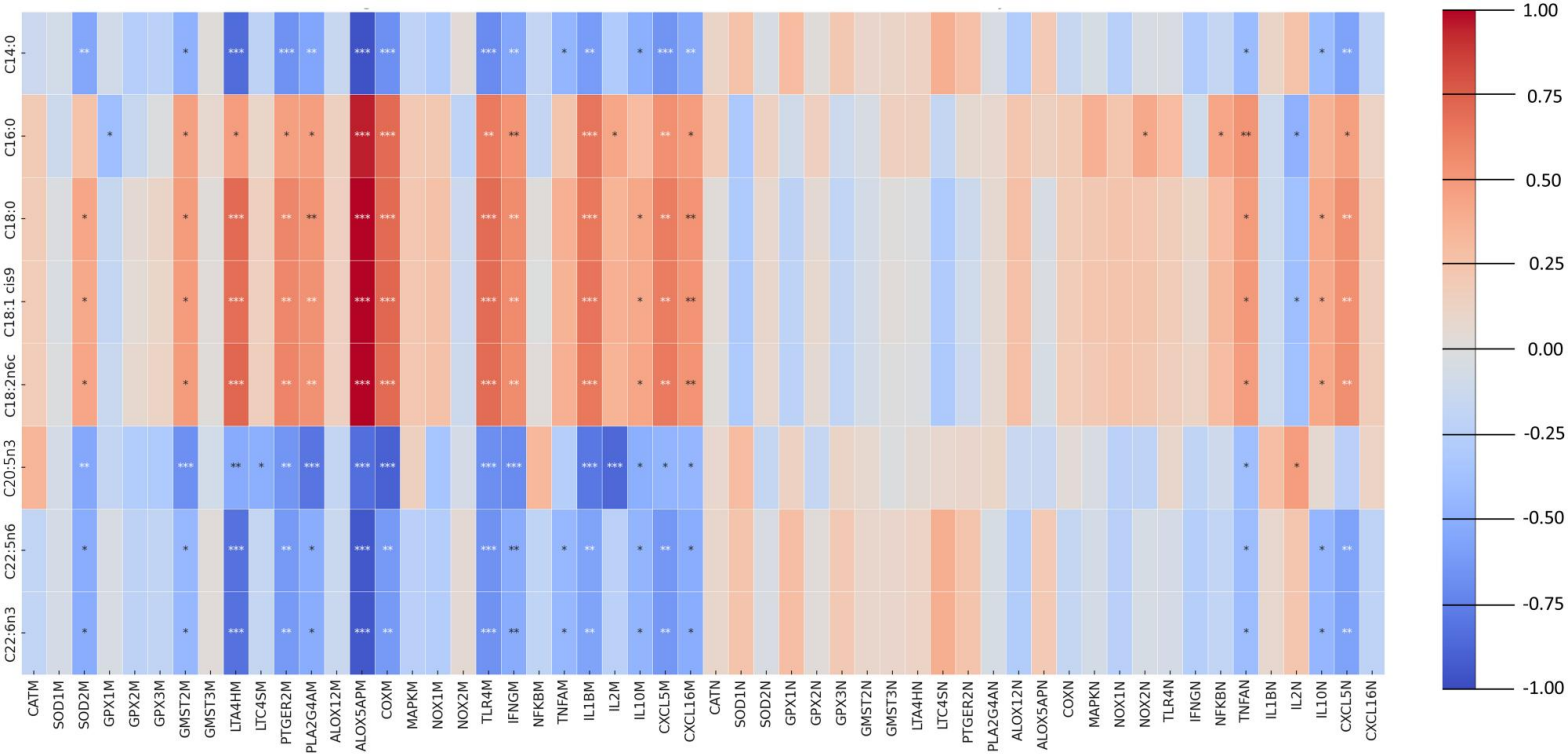
Pearson correlation of monocyte (M) and neutrophil (N) gene expression with principal fatty acid intake. Asterisks indicate levels of statistical significance (* *P* < 0.05, ** *P* < 0.01, *** *P* < 0.001).

The relationships between individual fatty acids intake and immune-oxidative gene expression were assessed using Pearson correlation analysis, and the results are summarized in Figure 5. Overall, the short- and medium-chain saturated fatty acids (C14:0, C16:0, C18:0) and monounsaturated C18:1 cis-9 displayed predominantly positive correlations with several pro-inflammatory and oxidative genes. In particular, C16:0 and C18:0 were strongly and positively associated with the expression of *ALOX5AP*, *COX*, *PTGER2*, *PLA2G4A*, *TLR4*, *IL1B*, *CCL5*, *CXCL16*, and *IFNG* (*R *> 0.30; *P *< 0.05) in monocytes, showing positive associations with genes involved in eicosanoid-related pathways. Similarly, linoleic acid (C18:2 n-6c) followed a comparable pattern, correlating positively with key inflammatory mediators, including *TLR4*, *NFκB*, and *IL1B*. In contrast, long-chain PUFA, particularly EPA, DPA, and DHA, showed predominantly negative correlations with pro-inflammatory genes. EPA exhibited the strongest negative correlations with pro-inflammatory mediators compared to DPA and DHA.

### Discriminant analyses

**Figure 6:**
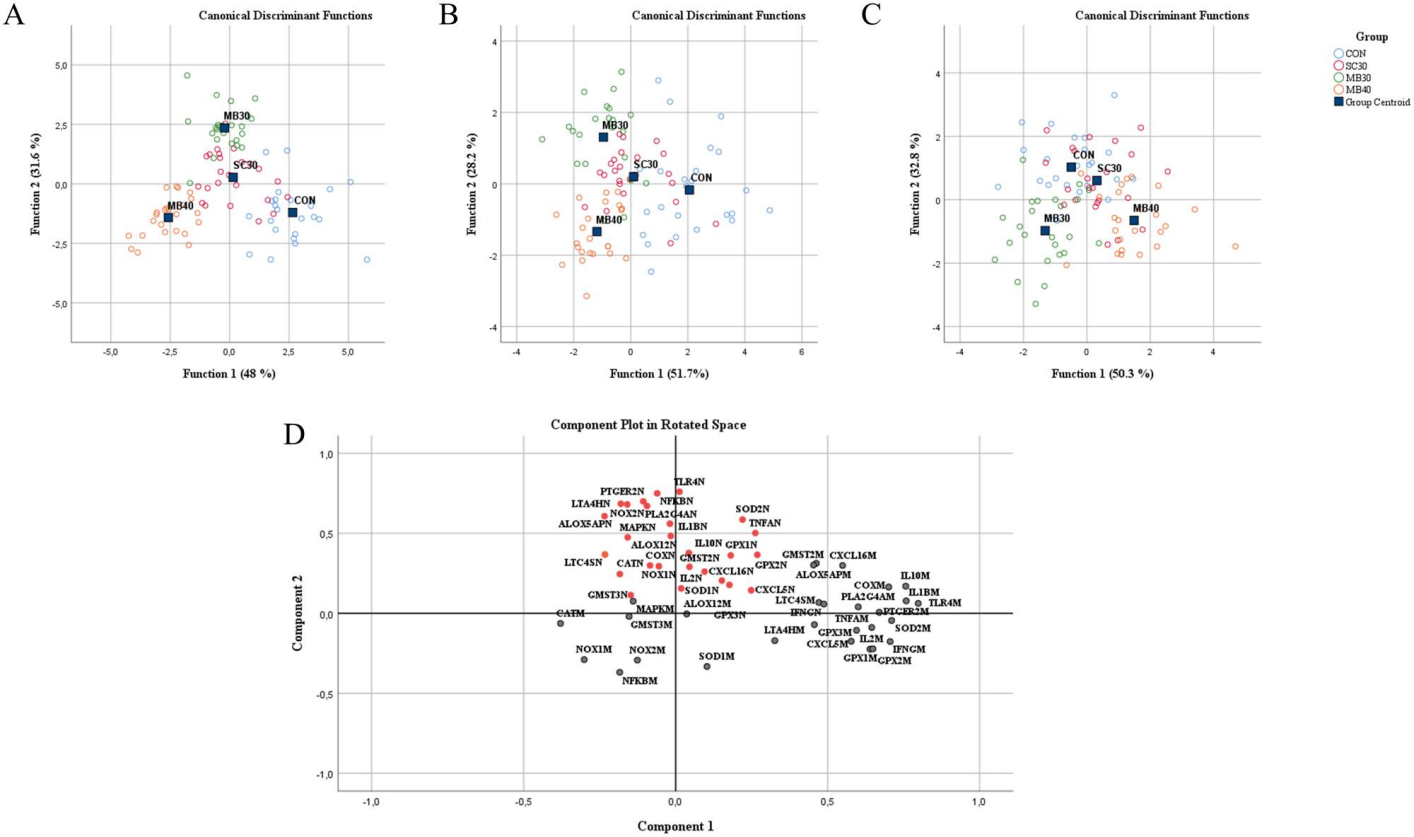
Discriminant plots showing the separation of the four dietary treatments (CON, SC30, MC30, and MB40) based on gene expression data from (A) both monocytes and neutrophils, (B) monocytes only, and (C) neutrophils only. (D) Principal component analysis (PCA) applied to combined monocyte and neutrophil gene expression profiles. Genes ending with “M” correspond to monocytes (black dots), whereas those ending with “N” correspond to neutrophils (red dots). CON: concentrate mix without microalgae; SC30: 30 g microalgae *Schizochytrium* spp.·animal^−1^·d^−1^; MB30: 30 microalgae blend g·animal^−1^·d^−1^ (21 g *Schizochytrium* spp. and 9 g *Nannochloropsis gaditana*); MB40: 40 microalgae blend g·animal^−1^·d^−1^ (28 g *Schizochytrium* spp. and 12 g *Nannochloropsis gaditana*).

In Figure 6A for both cell types, Function 1 explained the majority of between-group variance and was mainly characterized by genes expressed in monocytes related to eicosanoid synthesis and inflammatory signaling. The strongest positive loadings were observed for *ALOX5AP* (*R *= 0.317), *COX* (*R *= 0.269), *LTA4H* (*R *= 0.266), *PLA2G4A* (*R *= 0.243), *PTGER2* (*R *= 0.237), and *TLR4* (*R* = 0.233) in monocytes. Function 2 was defined by antioxidant and immune‐regulatory genes, with the highest absolute correlations for *GPX1* (*R *= 0.338), *GPX2* (*R *= 0.246) in monocytes, and negative loadings for *MAPK* (*R* = –0.209) and *NOX2* (*R* = –0.205) in neutrophils. This pattern suggests that Function 2 captured differences in oxidative–antioxidant balance across treatments, where higher microalgae inclusion (especially MB40). According to Wilks’ Lambda criterion, the discriminant functions were statistically significant (Function 1: Λ  =  0.026, *P *< 0.001; Function 2: Λ  =  0.117, *P *= 0.012). Together, the functions correctly classified 94.8% of the cases into their respective groups.

In Figure 6B for monocytes, Function 1 was dominated by monocyte genes involved in eicosanoid biosynthesis and innate immune activation, with the strongest positive loadings for *ALOX5AP* (*R *= 0.482), *COX* (*R *= 0.390), *IL1B* (*R *= 0.344), *TLR4* (*R *= 0.330), *PLA2G4A* (*R *= 0.310), and *PTGER2* (*R *= 0.292). Moderate contributions were also seen for *IFNG* and *GMST2*. These associations indicate that Function 1 primarily separated ewes according to activation of pro-inflammatory and lipid-mediator pathways. Function 2 was defined by genes encoding antioxidant enzymes and redox regulators, showing the highest correlations for *GPX1* (*R *= 0.576), *GPX2* (*R *= 0.465), and *SOD2* (*R *= 0.338), together with positive loadings for *LTA4H*, *GPX3*, *IL10*, and *SOD1*. According to Wilks’ Lambda criterion, both discriminant functions were statistically significant (Function 1: Λ  =  0.115, *P *< 0.001; Function 2: Λ  =  0.311, *P *< 0.001). Overall, 79.2% of the cases were correctly classified into their respective groups.

In Figure 6C for neutrophils, Function 1 was primarily defined by genes associated with pro-inflammatory and eicosanoid-related signaling. The strongest contributors were *IFNG* (*R* = –0.365), *PTGER2* (*R *= 0.288), *CAT* (*R *= 0.255), *LTA4H* (*R *= 0.248), *MAPK* (*R *= 0.246), and *ALOX5AP* (*R *= 0.244). These loadings suggest that Function 1 discriminated groups mainly based on inflammatory and oxidative response intensity, with CON and SC30 ewes showing higher activation of *IFNG* and *TLR4*-related signaling (also loadings are overlapped), whereas MB30 and MB40 displayed attenuated expressions. Function 2 reflected the immune-oxidative regulations, characterized by positive loadings for *TNFA* (*R *= 0.302), *NOX2* (*R *= 0.246), and negative correlations for *IL2* (*R* = –0.348) and *SOD1* (*R* = –0.202). According to Wilks’ Lambda criterion, Function 1 was statistically significant (Λ  =  0.198, *P *= 0.001), whereas Function 2 showed only a tendency toward significance (Λ  =  0.420, *P *= 0.057). Overall, 71.9% of the cases were correctly classified into their respective groups. Principal component analysis (Figure 6D) further discriminated the variables between monocytes and neutrophils, highlighting the distinct responses observed in the discriminant analysis.

## Discussion

### Nutrient intake

Feed and nutrient intake were not substantially affected by microalgae supplementation. In contrast, previous studies in goats reported reductions in feed intake when 40 or 60 g *Schizochytrium* spp.·animal^−1^·d^−1^ were included in the diet (Mavrommatis and Tsiplakou 2020; Mavrommatis et al. 2021c). Similarly, Papadopoulos et al. (2002) observed a 24.7% decrease in dry matter intake when ewes were supplemented with 47 g *Schizochytrium* spp.·animal^−1^·d^−1^. The absence of an effect in the present study may be attributed to the isocaloric and isolipidic formulation of the diets, where microalgae partially replaced plant fat and soybean oil, as well as to the lower inclusion levels of microalgae, particularly *Schizochytrium* spp. Given these considerations, the inclusion levels used in the present study were optimal, as no adverse effects on feed intake were observed.

### Antioxidant gene expression in monocytes and neutrophils

Dietary supplementation with microalgae rich in PUFA at high inclusion levels has been shown to induce oxidative stress through the activation of NADPH oxidase. More specifically, in our previous study, dietary supplementation of goats with 40 and 60 g *Schizochytrium* spp.·animal^−1^·d^−1^ increased the enzymatic activity of NADPH oxidase in blood plasma, as measured by native and SDS-PAGE (Mavrommatis et al. 2018). Additionally, in monocytes, the relative transcript levels of *NOX1* and *NOX2* tended to be upregulated with 40 and 60 g *Schizochytrium* spp.·animal^−1^·d^−1^ (Kyriakaki et al. 2023). In neutrophils, both *NOX1* and *NOX2* were significantly upregulated only at 60 g *Schizochytrium* spp.·animal^−1^·d^−1^, clearly demonstrating the importance of inclusion level in oxidative manifestation (Kyriakaki et al. 2023). These upregulation of superoxide anion generators diversely affect antioxidant mechanisms since *SOD2* and *SOD3* in monocytes and *GMST1* in neutrophils were downregulated while the activity of plasmatic SOD was increased (Mavrommatis et al. 2018; Kyriakaki et al. 2023) pointing out a remodeling of the antioxidant mechanisms. In the present study, the lower level of PUFA supplementation in the ewes’ diet, combined with the simultaneous inclusion of *Schizochytrium* spp. and *Nannochloropsis gaditana*, downregulated *NOX2* expression in neutrophils (MB30 group), while sustaining unaffected *NOX1* expression in both cell types. Based on this set of evidence, it can be concluded that the microalgae levels in all dietary treatments, and particularly in the MB30 group, were optimal to prevent the activation of the prevailing ROS-generating system, at least at transcriptional level. However, *CAT* expression in SC30 monocytes and MB30 neutrophils was downregulated, whereas *SOD2*, *GPX1*, *GPX2*, *GPX3*, and *GMST2* collectively exhibited a suppressed trend in the MB40-monocytes, which corresponded to the highest inclusion level of microalgae. Superoxide dismutase (SOD) constitutes the first line of defence against oxidative stress, protecting cells from damage by detoxifying the superoxide anion, while SOD2 represents the mitochondrial isoform of the enzyme (Palma et al. 2020). Catalase (CAT) and glutathione peroxidase (GSH-Px) form the second line of defence by catalyzing the conversion of hydrogen peroxide to water and oxygen, and the reduction of peroxides to alcohols and water (Jomova et al. 2023). In agreement with our findings, Rudkowska et al. (2013) observed an approximately 17% decrease in *GMST1* expression following dietary supplementation with n-3 PUFAs in humans.

The aforementioned antioxidant remodeling at transcriptional level raises questions regarding the occurrence of pro-oxidant events and the potential involvement of an alternative pathway to NADPH oxidase activation, or a systemic downregulation of endogenous antioxidant mechanisms induced by the administration of exogenous antioxidant compounds derived from microalgae. This interpretation is supported by the observed downregulation of several second-step detoxification enzymes (CAT, GPX), likely reflecting reduced endogenous antioxidant demand due to both the inhibition of *NOX* and the presence of exogenous antioxidants derived from microalgae acting extracellularly or in the cytosol. On the other hand, the observed downregulation of *SOD2* in monocytes may reflect a reduced mitochondrial ROS load secondary to the anti-inflammatory and antioxidant properties of microalgae. Given that *SOD2* expression is inducible by oxidative and cytokine stimuli, the lower pro-inflammatory status observed in the present study could have decreased mitochondrial ROS signaling requirements, thereby reducing the need for SOD2-mediated antioxidant defense. However, at this stage, it is difficult to draw reliable conclusions about the mechanisms underlying the antioxidant remodeling observed in the MB40 group, or whether these transcriptional changes are reflected at the protein or functional level.

In our previous study, we concluded that supplementation providing more than 70 mg of DHA and DPA from *Schizochytrium* spp./kg BW in small ruminants can induce excessive ROS production through NOX activation, thereby impairing antioxidant mechanisms (Mavrommatis et al. 2018; Kyriakaki et al. 2023). In the present study, although this upper bound was followed, *NOX* gene expression was not elevated in either cell type; nevertheless, alterations in antioxidant gene expression were observed. It is worth noting, however, that while our individual analyses did not provide sufficient resolution to clearly identify a pro-oxidant stress response in the MB40 group based on *NOX* transcript levels across cell types, Pearson correlation analysis revealed a strong intercellular association (negative correlation), between neutrophilic *NOX* relative abundance and monocyte antioxidant gene expression, suggesting a cross-cellular regulatory configuration that may share both cause and consequence.

### Effects on immune-related gene expression

Monocytes and neutrophils utilize fatty acids to produce eicosanoids, lipid mediators that orchestrate inflammation by modulating cytokine production and immune cell activity (Calder 2001). Dietary long-chain PUFA are incorporated into immune cell membranes, while EPA, DPA, and DHA substitute ARA, the predominant phospholipid PUFA and main precursor of eicosanoid synthesis under basal feeding conditions in ruminants. Notably, fish oil (also rich in EPA and DHA) dietary inclusion in human and animal models decreased the proportion of ARA in immune cell membranes, while the concentrations of EPA and DHA were increased (Calder 2015). These long-chain PUFA are detached from the phospholipid membrane through the action of A2 phospholipase (PLA2G4A) and used for eicosanoid production under a complex network of enzymes (Calder 2015). In our study, *PLA2G4A* mRNA levels in monocytes were not affected by *Schizochytrium* spp. inclusion (SC30) but were significantly altered with the microalgae blend (MB30 and MB40). This suggests that the regulation of *PLA2G4A* is influenced not solely by the total level of long-chain PUFA supplementation but more importantly by the complementary interactions among individual fatty acids, since 4.82, 3.70, and 4.91 g·d^−1^ of EPA, DPA, and DHA were supplied in the SC30, MB30, and MB40 ewes, respectively. This advantage of EPA over DHA may be attributed to its faster incorporation into cell membranes before being detached and utilized for eicosanoid production (Calder 2018). This hypothesis is further supported by the correlation between fatty acid intake and gene expression, where dietary EPA showed the strongest association with *PLA2G4A* expression in monocytes, compared with DHA and DPA, despite the latter being present at higher levels. In accordance with our findings, in a previous study carried out by Smesny et al. (2014), n-3 compared to n-6 PUFA dietary supplementation in humans decreased the activity of A2 phospholipase. However, attributing the observed correlations solely to the fatty acid content of microalgae may overlook the potential co-influence of other bioactive compounds present in microalgae.

Pro-resolving lipid mediators are biosynthesized by the sequential actions of 15-LOX (ALOX15), 12-LOX (ALOX12), and 5-LOX (ALOX5) on DHA, DPA, and ARA, while the potent chemoattractant and pro-inflammatory mediator LTB4 is synthesized from ARA by 5-LOX (ALOX5) acting with 5-LOX activating protein (ALOX5AP), followed by LTA4 hydrolase (LTA4H) (Orr et al. 2015). *ALOX5AP* was significantly downregulated in monocytes for all microalgae-fed treatments and for MB30-neutrophils while *LTA4H* was decreased only in monocytes and more intensively in SC30 and MB40 where *Schizochytrium* spp. was in the higher inclusion levels. This is also imprinted by the correlation between fatty acid intake and gene expression, where DHA and DPA showed the strongest correlation with *LTA4H* expression in monocytes, compared with EPA. The inhibition of the 5-LOX pathway might be explained by the lower availability of ARA in neutrophil and monocyte membranes in microalgae-fed goats, since DHA and EPA antagonize ARA for lipoxygenase and cyclooxygenase active sites (Calder 2001). Monocytes from the microalgae-treated groups, with greater emphasis on the highest combination of the two microalgae (MB40), showed a downregulation of cyclooxygenase-2 (PTGS2/COX) genes relative to the CON group. This suggests a lower capacity to synthesize pro-inflammatory eicosanoids like PGE_2_ from ARA in the presence of ample n-3 like DHA and EPA. Also DHA can give rise to resolvins and protectins, which actively resolve inflammation. Taken together, microalgal n-3 fatty acids were associated with modulation of eicosanoid-related gene expression at the transcriptional level, potentially contributing to less pronounced inflammatory signaling in treated monocytes and neutrophils compared with controls. Similar outcomes have been reported in macrophages from mice fed diets enriched with EPA and DHA, as well as in neutrophils from humans supplemented with marine n-3 fatty acids for several weeks (Calder 2015). Lastly, within the context of immune–oxidative crosstalk, resolvins and other DHA-derived eicosanoids produced through COX-mediated pathways can suppress superoxide anion generation. This mechanism may partly account for the observed modulation of antioxidant gene expression, reflecting a lower cellular requirement for redox regulation (Kohli and Levy 2009).

The regulatory properties of microalgae on eicosanoid production are associated with a shift toward a lower grade pro-inflammatory gene expression profile relative to the CON. Notably, pro-inflammatory cytokine genes (such as *IFNG*, *IL1B*, and *IL2*) were downregulated in monocytes of microalgae supplemented animals. This suggests that DHA, EPA, and DPA might attenuate the monocyte-driven inflammatory responses at transcriptional level. Many in vitro studies have shown a significant downregulation of the prevailing proinflammatory cytokines, IL1B and TNF, in human monocytes and rats macrophages treated with fish oil, rich in EPA and DHA, showing a reduction in pro-inflammatory response (Sinha et al. 1991; Baldie et al. 1993; Yaqoob and Calder 1995; Wallace et al. 2000). Further to the previous regulatory mechanisms, the downregulation of pro-inflammatory cytokines and chemokines may attribute to the direct or indirect effects of PUFA on pattern recognition receptors, and especially the well documented TLR4. TLR4 primarily recognizes LPS, a major component of the outer membrane of Gram-negative bacteria, and secondarily responds to viral components and heat shock proteins, while its stimulation mainly leads to cytokine and chemokine production (Oh et al. 2010). This overall pro-inflammatory downregulation may be attributed to the increased long-chain PUFA content of microalgae which could inhibit TLR4 inflammatory signaling through the G-protein coupled receptor 120 (GPR120) (Oh et al. 2010). GPR120 regulates the TLR4 pathway either indirectly via the inhibition of ІΚΚβ kinase phosphorylation or directly along with LPS binding failure by TLRs (Oh et al. 2010). Indeed, human macrophages and B cells treated with DHA showed a suppressed expression level of *TLR4*, while *GPR120* expression was upregulated (Radzikowska et al. 2019). Additionally, in our previous study supplementing goats diet with 20, 40, and 60 g *Schizochytrium* spp.·animal^−1^·d^−1^, *TLR4* was significantly downregulated in all treatment in monocytes compared to the control group. Controlling excessive inflammatory responses in intensively bred ruminants is of substantial practical relevance, as nutrients are strongly partitioned between the metabolic demands of production and those required to sustain immune function (Kvidera et al. 2017; McGrath et al. 2018). Nevertheless, it is essential that attenuation of the inflammatory response does not compromise immune competence, as ruminants raised under intensive systems are continuously exposed to environmental and metabolic challenges that require a responsive and functional immune system. In our study, the downregulation of pro-inflammatory genes was accompanied by a parallel decrease in *IL10*, the principal anti-inflammatory regulator, suggesting that the immune system was already in a quiescent state, with reduced need for *IL10*-driven resolution. This coordinated reduction likely reflects balanced immune modulation rather than immunosuppression. However, further experimentation, including immune challenge trials and extended study durations, should be conducted to thoroughly evaluate any potential adverse effects on immune system readiness.

The outcomes of this study differ in part from those previously reported in human THP-1 macrophages regarding the relative anti-inflammatory potential of individual PUFA. Specifically, Weldon et al. (2007) reported that DHA induces an anti-inflammatory profile in LPS-stimulated THP-1 macrophages more effectively than EPA. In contrast, our results demonstrated that EPA, even when present at lower concentrations compared to other PUFA, more strongly downregulated eicosanoid production and, consequently, cytokine expression. Alternatively, these effects may reflect the complementary and synergistic actions of long-chain PUFA, which may enhance the immunomodulatory properties of microalgae. The former hypothesis have been previously reported, since dietary inclusion of *Schizochytrium* spp. (40% DHA, 16% DPA) in rats reduced the overall inflammation due to the stronger effect of the synergistic action of these PUFA compared to DHA per se (Nauroth et al. 2010). Although the DPA contained in *Schizochytrium* spp. is an n-6 isomer, it is important to note that not all n-6 PUFA exert pro-inflammatory effects, contrary to the long-held view. Previous studies have shown that n-6 DPA can be more effective than either DHA or EPA alone in reducing pro-inflammatory cytokine production in vitro, and that in vivo, orally administered n-6 DPA may enhance the anti-inflammatory action of DHA during acute inflammation (Dangi et al. 2010). Additionally, beyond the potential synergistic effects of PUFA contained in microalgae, their combination may exert more pronounced effects due to the presence of numerous bioactive compounds other than lipids.

### Differential responses between monocytes and neutrophils

Despite both cell types responding to microalgae supplementation, monocytes and neutrophils exhibited distinct response profiles in relation to DHA, DPA, and EPA intake, which helps explain the differences observed between treated and control groups. Monocytes, being longer-lived circulating precursors to macrophages, showed more pronounced changes at the mRNA level. Kyriakaki et al. (2023) and Mavrommatis et al. (2021a) reported that monocytes had stronger alterations of antioxidant and pro-inflammatory genes than neutrophils did. In fact, Rico et al. (2017) suggest a greater plasticity of monocyte epigenomes compared to neutrophils. Monocytes also incorporated a higher proportion of DHA/EPA into their cell membranes, which was reflected in greater shifts in their cytokine secretion profiles (Calder 2018). In contrast, neutrophils, which are short-lived first responders, displayed more modest gene expression changes but significant functional alterations. The observed differences may be attributed to inherent cellular properties, as monocytes exhibit greater transcriptional activity and phenotypic plasticity, enabling them to respond more markedly to microalgae supplementation, resulting in an anti-inflammatory and antioxidant transcriptional profile. Neutrophils, on the other hand, rely on pre-formed components for their rapid responses; n-3 fatty acids likely exert effects by altering cell membrane raft composition and receptor signaling rather than broad gene induction (Yaqoob 2003). Consequently, while both cell types benefit from microalgal n-3 by reduced inflammatory tendencies, monocytes show larger changes in gene expression.

## Conclusions

By combining Schizochytrium spp. and Nannochloropsis gaditana, two complementary sources of long-chain n-3 fatty acids, dietary supplementation was associated with changes in redox- and immune-related gene expression in circulating leukocytes, without affecting feed intake. The findings indicate that microalgae supplementation is associated with modulation of cellular transcriptional responses. Collectively, these outcomes highlight the potential of microalgae as feed additives within the framework of immunonutrition. Future studies should investigate whether these molecular alterations translate into measurable changes in immune responsiveness, oxidative status, and animal performance.

## Supplementary Material

skag165_Supplementary_Data

## Data Availability

The data underlying this article are available in the article and in its online supplementary material..

## Abbreviations

ALOX5AP: arachidonate 5-lipoxygenase–activating protein
ALOX15: arachidonate 15-lipoxygenase
ARA: arachidonic acid
CAT: catalase
COX: cyclooxygenase (prostaglandin-endoperoxide synthase 2)
CXCL5: C-X-C motif chemokine ligand 5
CXCL16: C-X-C motif chemokine ligand 16
DHA: docosahexaenoic acid
DPA: docosapentaenoic acid
EPA: eicosapentaenoic acid
GAPDH: glyceraldehyde 3-phosphate dehydrogenase
GPX1: glutathione peroxidase 1
GPX2: glutathione peroxidase 2
GPX3: glutathione peroxidase 3
GPR120: G-protein–coupled receptor 120
GMST2: glutathione S-transferase 2
GMST3: glutathione S-transferase 3
IFNG: interferon gamma
IL1B: interleukin 1 beta
IL2: interleukin 2
IL10: interleukin 10
LTA4H: leukotriene A4 hydrolase
LTC4S: leukotriene C4 synthase
MAPK: mitogen-activated protein kinase
NFKB: nuclear factor kappa-light-chain–enhancer of activated B cells
NOX1: NADPH oxidase 1
NOX2: NADPH oxidase 2
PLA2G4A: phospholipase A2 group IVA
PTGER2: prostaglandin E receptor 2
PUFA: polyunsaturated fatty acid
ROS: reactive oxygen species
SOD1: superoxide dismutase 1
SOD2: superoxide dismutase 2
TLR4: toll-like receptor 4
TNFA: tumor necrosis factor alpha
YWHAZ: tyrosine 3-monooxygenase/tryptophan 5-monooxygenase activation protein zeta
