# Supplementary material for: Dietary microalgae Schizochytrium spp. and Nannochloropsis gaditana modulate antioxidant and immune-related gene expression in ewes’ monocytes and neutrophils
Source: J Anim Sci. 2026 May 22;104:skag165. doi: 10.1093/jas/skag165 (PMC13257863; doi:10.1093/jas/skag165)
Supplement: skag165_Supplementary_Data [file skag165_supplementary_data.docx]

**Supplementary Table 1.** Concentrate ingredient composition.

|  | CON | SC30 | MB30 | MB40 |
| --- | --- | --- | --- | --- |
| Item (in g/kg as fed) | | | | |
| Maize grain | 472.0 | 469.5 | 469.5 | 464.5 |
| Sunflower meal | 80 | 80 | 80 | 80 |
| Wheat middling | 70 | 70 | 70 | 70 |
| Barley | 100 | 100 | 100 | 100 |
| Soyabean meal | 185 | 185 | 185 | 185 |
| Calcium carbonate | 14 | 14 | 14 | 14 |
| ^1^Sodium bicarbonate | 3 | 3 | 3 | 3 |
| ^2^Premix of minerals and vitamins | 25 | 25 | 25 | 25 |
| ^3^Mycotoxin binder | 1 | 1 | 1 | 1 |
| Dried brewer’s yeast | 25 | 25 | 25 | 25 |
| Soyabean oil | 5 | 2.5 | 2.5 | 2.5 |
| ^4^Palm oil | 20 | 10 | 10 | 10 |
| ^5^*Schizochytrium spp.* | - | 15 | 10.5 | 14 |
| ^6^*Nannochloropsis gaditana* | - | - | 4.5 | 6 |

CON: concentrate mix without microalgae; SC30: 30 g microalgae Schizochytrium spp./head/day; MB30: 30 microalgae blend g/head/day (21 g Schizochytrium spp. and 9 g Nannochloropsis gaditana); MB40: 40 microalgae blend g/head/day (28 g Schizochytrium spp. and 12 g Nannochloropsis gaditana).

^1^Bicar Z 0/50 Sodium Bicarbonate

^2^Premix of minerals and vitamins provides, per kg of product: vitamin A (E672), 480,000 IU; vitamin D₃ (E671), 80,000 IU; vitamin E (E307), 1,200 IU; iodine (as calcium iodate, anhydrous), 40 mg; cobalt (as cobalt sulfate), 12 mg; niacin, 800 mg; iron (as iron carbonate), 2,400 mg; manganese (as manganese oxide), 1,600 mg; selenium (as sodium selenite), 8 mg; zinc (as chelated zinc), 300 mg. An aromatic additive (LUCTAROM) is also included.

^3^TOXFIN® Dry based on specially activated aluminosilicate components.

^4^Bergafat T-300 is derived from fractionated palm oil and contains its natural glycerol.

^5^DHAgold™ (DSM; DHAgold S17-B).

^6^Cultivated for the needs of the trial by Mikrophykos, Athens, Greece.

**Supplementary Table 2.** Chemical composition and fatty acid profile of *Schizochytrium spp.* and *Nannochloropsis gaditana.*

|  | *Schizochytrium spp.* | *Nannochloropsis gaditana* |
| --- | --- | --- |
| Item as g/kg as fresh |  |  |
| Dry matter | 980 | 915 |
| Crude protein | 167 | 410 |
| Ether extract | 556 | 100 |
| Ash | 88 | 100 |
| % of total fatty acids |  |  |
| C14:0 | 5.9 | 4.5 |
| C16:0 | 13.1 | 18.3 |
| C18:0 | 0.30 | 0.2 |
| cis-9 C18:1 | - | 4.7 |
| C18:3 n-3 | 0.1 | - |
| C20:5 n-3 | - | 34.5 |
| C22:5 n-6 | 6.8 | - |
| C22:6 n-3 | 21.8 | - |

**Supplementary Table 3**. Repeated-measures analysis of variance (RM-ANOVA) for antioxidant-, eicosanoid-, and immune-related gene expression in neutrophils.

| **Gene** | **CON** | **SC30** | **MB30** | **MB40** | **30 DAY** | **60 DAY** | **90 DAY** | **SEM** | **D** | **T** | **D x T** |
| --- | --- | --- | --- | --- | --- | --- | --- | --- | --- | --- | --- |
| *CAT* | 1.000^ab^ | 0.992^ab^ | 0.789^a^ | 1.177^b^ | 1.120^Α^ | 0.983^ΑΒ^ | 0.865^Β^ | 0.051 | 0.028 | 0.025 | 0.000 |
| *SOD1* | 1.000 | 1.199 | 1.415 | 1.402 | 1.143^A^ | 0.801^B^ | 1.818^C^ | 0.099 | 0.247 | 0.001 | 0.001 |
| *SOD2* | 1.000 | 1.036 | 0.803 | 0.894 | 0.921 | 0.828 | 1.051 | 0.090 | 0.850 | 0.398 | 0.029 |
| *GPX1* | 1.000^a^ | 1.388^ab^ | 0.988^a^ | 1.590^b^ | 0.829^B^ | 0.934^B^ | 1.962^A^ | 0.127 | 0.066 | 0.001 | 0.002 |
| *GPX2* | 1.000 | 1.110 | 0.707 | 0.992 | 0.582^B^ | 0.741^B^ | 1.535^A^ | 0.096 | 0.262 | <0.001 | 0.021 |
| *GPX3* | 1.000 | 1.386 | 1.145 | 1.508 | 0.520^B^ | 1.038^B^ | 2.221^A^ | 0.164 | 0.562 | <0.001 | 0.010 |
| *GMST2* | 1.000 | 1.057 | 1.019 | 1.132 | 1.177 | 0.976 | 1.003 | 0.078 | 0.968 | 0.236 | 0.088 |
| *GMST3* | 1.000 | 1.054 | 0.750 | 1.175 | 1.327^A^ | 0.775^B^ | 0.882^B^ | 0.070 | 0.360 | <0.001 | 0.136 |
| *LTA4H* | 1.000 | 1.047 | 0.736 | 1.247 | 1.139 | 1.082 | 0.800 | 0.071 | 0.136 | 0.104 | 0.484 |
| *LTC4S* | 1.000 | 1.510 | 1.094 | 1.483 | 1.273 | 1.359 | 1.184 | 0.103 | 0.335 | 0.618 | 0.040 |
| *PTGER2* | 1.000^ab^ | 1.173^ab^ | 0.788^a^ | 1.371^b^ | 1.087 | 1.033 | 1.128 | 0.075 | 0.042 | 0.865 | 0.151 |
| *PLA2G4A* | 1.000 | 0.889 | 0.918 | 1.059 | 1.006 | 0.991 | 0.903 | 0.060 | 0.623 | 0.790 | 0.166 |
| *ALOX12* | 1.000 | 0.809 | 0.849 | 0.803 | 0.924 | 0.944 | 0.728 | 0.057 | 0.792 | 0.125 | 0.247 |
| *ALOX5AP* | 1.000^ab^ | 1.234^a^ | 0.717^b^ | 1.175^a^ | 1.347^A^ | 0.944^B^ | 0.803^B^ | 0.071 | 0.098 | 0.002 | 0.414 |
| *COX* | 1.000 | 0.822 | 0.843 | 0.978 | 0.800 | 1.025 | 0.907 | 0.056 | 0.716 | 0.132 | 0.020 |
| *MAPK* | 1.000^b^ | 0.914^ab^ | 0.736^a^ | 1.056^b^ | 0.930^AB^ | 1.033^A^ | 0.816^B^ | 0.040 | 0.046 | 0.065 | 0.456 |
| *NOX1* | 1.000 | 0.876 | 0.831 | 0.790 | 0.772 | 0.929 | 0.922 | 0.057 | 0.772 | 0.146 | 0.018 |
| *NOX2* | 1.000^b^ | 0.966^b^ | 0.605^a^ | 1.015^b^ | 0.976^A^ | 0.955^AB^ | 0.759^B^ | 0.055 | 0.020 | 0.029 | 0.001 |
| *TLR4* | 1.000^ab^ | 0.829^ab^ | 0.666^a^ | 1.145^b^ | 0.879^B^ | 0.874^B^ | 0.97^A^ | 0.061 | 0.089 | 0.028 | 0.003 |
| *IFNG* | 1.000^a^ | 0.908^ab^ | 1.228^a^ | 0.604^b^ | 0.637 | 0.909 | 1.258 | 0.063 | 0.024 | 0.101 | 0.538 |
| *NFKB* | 1.000^a^ | 0.898^ab^ | 0.780^b^ | 0.990^a^ | 0.987^A^ | 0.917^AB^ | 0.847^B^ | 0.032 | 0.080 | 0.011 | 0.002 |
| *TNFA* | 1.000^a^ | 0.818^ab^ | 0.680^b^ | 0.762^ab^ | 0.812^A^ | 0.836^A^ | 0.796^B^ | 0.044 | 0.072 | 0.057 | 0.018 |
| *IL1B* | 1.000 | 0.944 | 1.109 | 1.236 | 1.246^A^ | 0.943^B^ | 1.029^AB^ | 0.074 | 0.763 | 0.039 | 0.075 |
| *IL2* | 1.000^b^ | 1.084^ab^ | 1.516^a^ | 1.304^ab^ | 1.129^AB^ | 1.102^B^ | 1.447^A^ | 0.070 | 0.098 | 0.073 | 0.566 |
| *IL8* | 1.000 | 1.185 | 1.614 | 3.016 | 0.222^A^ | 0.878^B^ | 4.010^C^ | 0.386 | 0.167 | 0.001 | 0.003 |
| *IL10* | 1.000^a^ | 0.656^b^ | 0.810^ab^ | 0.885^ab^ | 0.985^A^ | 0.801^B^ | 0.727^B^ | 0.045 | 0.099 | 0.039 | 0.549 |
| *CXCL5* | 1.000^a^ | 0.654^b^ | 0.761^ab^ | 0.687^b^ | 0.749 | 0.820 | 0.757 | 0.047 | 0.097 | 0.771 | 0.772 |
| *CXCL16* | 1.000 | 0.844 | 0.949 | 0.983 | 0.955 | 1.008 | 0.870 | 0.036 | 0.626 | 0.128 | 0.108 |

Effects of diet (D), sampling time (S), and their interaction (D × S) are presented as P-values. Statistical significance was declared at P < 0.05, while 0.05 ≤ P < 0.10 was considered a statistical trend. Different lowercase superscript letters indicate differences among dietary treatments, whereas different uppercase superscript letters indicate differences due to sampling time. CON: concentrate mix without microalgae; SC30: 30 g microalgae *Schizochytrium spp*./head/day; MB30: 30 microalgae blend g/head/day (21 g *Schizochytrium spp*. and 9 g *Nannochloropsis gaditana*); MB40: 40 microalgae blend g/head/day (28 g *Schizochytrium spp*. and 12 g *Nannochloropsis gaditana*).

**Supplementary Table 4**. Repeated-measures analysis of variance (RM-ANOVA) for antioxidant-, eicosanoid-, and immune-related gene expression in monocytes.

| **Gene** | **CON** | **SC30** | **MB30** | **MB40** | **30 DAY** | **60 DAY** | **90 DAY** | **SEM** | **D** | **T** | **D x T** |
| --- | --- | --- | --- | --- | --- | --- | --- | --- | --- | --- | --- |
| *CAT* | 1.000^a^ | 0.740^b^ | 0.867^ab^ | 1.111^a^ | 1.007^A^ | 1.007^A^ | 0.774^B^ | 0.044 | 0.028 | 0.033 | 0.002 |
| *SOD1* | 1.000 | 1.012 | 1.166 | 0.840 | 0.939 | 1.055 | 1.019 | 0.061 | 0.452 | 0.570 | 0.001 |
| *SOD2* | 1.000^a^ | 0.807^a^ | 0.910^a^ | 0.389^b^ | 0.514^A^ | 0.733^A^ | 1.082^B^ | 0.060 | 0.001 | <0.001 | 0.302 |
| *GPX1* | 1.000^c^ | 1.191^c^ | 1.647^b^ | 0.554^a^ | 0.700^A^ | 1.006^B^ | 1.588^C^ | 0.083 | <0.001 | <0.001 | 0.000 |
| *GPX2* | 1.000^c^ | 1.003^c^ | 1.378^b^ | 0.404^a^ | 0.432^A^ | 1.051^B^ | 1.356^C^ | 0.086 | <0.001 | <0.001 | 0.000 |
| *GPX3* | 1.000^ab^ | 0.992^ab^ | 1.122^a^ | 0.541^b^ | 0.844 | 0.922 | 0.975 | 0.081 | 0.094 | 0.777 | 0.242 |
| *GMST2* | 1.000^a^ | 0.904^a^ | 0.741^ab^ | 0.571^b^ | 0.894 | 0.732 | 0.786 | 0.047 | 0.049 | 0.180 | 0.089 |
| *GMST3* | 1.000 | 1.053 | 0.897 | 1.004 | 1.149^A^ | 1.044^A^ | 0.772^B^ | 0.068 | 0.935 | 0.021 | 0.018 |
| *LTA4H* | 1.000^a^ | 0.700^b^ | 0.861^c^ | 0.550^d^ | 0.760 | 0.817 | 0.757 | 0.036 | <0.001 | 0.658 | 0.217 |
| *LTC4S* | 1.000^a^ | 1.095^a^ | 0.956^ab^ | 0.558^b^ | 0.860 | 0.915 | 0.931 | 0.069 | 0.098 | 0.849 | 0.748 |
| *PTGER2* | 1.000^a^ | 0.730^b^ | 0.738^b^ | 0.340^c^ | 0.511^A^ | 0.684^AB^ | 0.911^B^ | 0.058 | 0.003 | 0.008 | 0.266 |
| *PLA2G4A* | 1.000^a^ | 0.891^ab^ | 0.699^b^ | 0.352^c^ | 0.536^A^ | 0.818^B^ | 0.852^B^ | 0.058 | <0.001 | 0.018 | 0.208 |
| *ALOX12* | 1.000 | 0.943 | 0.895 | 0.859 | 1.017^A^ | 1.053^A^ | 0.703^B^ | 0.056 | 0.829 | 0.044 | 0.075 |
| *ALOX5AP* | 1.000^a^ | 0.573^b^ | 0.453^b^ | 0.350^b^ | 0.555 | 0.712 | 0.515 | 0.047 | <0.001 | 0.124 | 0.248 |
| *COX* | 1.000^a^ | 0.802^ab^ | 0.509^b^ | 0.331^b^ | 0.427^A^ | 0.799^B^ | 0.755^B^ | 0.056 | 0.002 | <0.001 | 0.077 |
| *MAPK* | 1.000 | 0.844 | 0.916 | 0.998 | 0.980 | 0.946 | 0.892 | 0.031 | 0.276 | 0.492 | 0.327 |
| *NOX1* | 1.000 | 0.912 | 0.874 | 0.703 | 0.914^A^ | 1.175^B^ | 0.528^C^ | 0.055 | 0.271 | <0.001 | 0.008 |
| *NOX2* | 1.000 | 1.114 | 1.188 | 0.923 | 1.077^A^ | 1.244^A^ | 0.848^B^ | 0.053 | 0.233 | 0.002 | 0.000 |
| *TLR4* | 1.000^a^ | 0.738^b^ | 0.652^b^ | 0.492^c^ | 0.492^A^ | 0.602^B^ | 1.068^C^ | 0.046 | 0.003 | 0.008 | 0.250 |
| *IFNG* | 1.000^a^ | 0.788^ab^ | 0.566^bc^ | 0.322^c^ | 0.271^A^ | 0.780^B^ | 0.955^B^ | 0.069 | 0.010 | <0.001 | 0.009 |
| *NFKB* | 1.000^ab^ | 0.860^a^ | 1.125^b^ | 0.981^ab^ | 1.049 | 1.012 | 0.914 | 0.030 | 0.022 | 0.187 | 0.000 |
| *TNFA* | 1.000 | 0.811 | 0.927 | 0.726 | 0.737^A^ | 0.949^B^ | 0.912^B^ | 0.041 | 0.285 | 0.005 | 0.000 |
| *IL1B* | 1.000^a^ | 0.778^ab^ | 0.480^bc^ | 0.337^c^ | 0.399^A^ | 0.482^A^ | 1.065^B^ | 0.062 | 0.001 | <0.001 | 0.241 |
| *IL2* | 1.000^a^ | 1.174^a^ | 0.551^b^ | 0.359^b^ | 0.448^A^ | 0.816^B^ | 1.050^B^ | 0.075 | 0.001 | 0.003 | 0.009 |
| *IL8* | 1.000 | 1.017 | 0.693 | 0.285 | 0.579 | 0.756 | 0.910 | 0.151 | 0.427 | 0.381 | 0.003 |
| *IL10* | 1.000^a^ | 0.860^ab^ | 0.883^ab^ | 0.605^b^ | 0.683^A^ | 0.879^B^ | 0.948^B^ | 0.045 | 0.098 | 0.002 | 0.103 |
| *CXCL5* | 1.000^a^ | 0.619^ab^ | 0.650^ab^ | 0.487^b^ | 0.567^B^ | 0.691 ^AB^ | 0.809 ^A^ | 0.057 | 0.100 | 0.077 | 0.005 |
| *CXCL16* | 1.000 | 0.809 | 0.774 | 0.699 | 0.812 | 0.855 | 0.794 | 0.039 | 0.199 | 0.714 | 0.084 |

Effects of diet (D), sampling time (S), and their interaction (D × S) are presented as P-values. Statistical significance was declared at P < 0.05, while 0.05 ≤ P < 0.10 was considered a statistical trend. Different lowercase superscript letters indicate differences among dietary treatments, whereas different uppercase superscript letters indicate differences due to sampling time. CON: concentrate mix without microalgae; SC30: 30 g microalgae *Schizochytrium spp*./head/day; MB30: 30 microalgae blend g/head/day (21 g *Schizochytrium spp*. and 9 g *Nannochloropsis gaditana*); MB40: 40 microalgae blend g/head/day (28 g *Schizochytrium spp*. and 12 g *Nannochloropsis gaditana*).
